# Supplementary material for: Static and dynamic fMRI-derived functional connectomes represent largely similar information
Source: Netw Neurosci. 2023 Dec 22;7(4):1266–301. doi: 10.1162/netn_a_00325 (PMC10631791; doi:10.1162/netn_a_00325)
Supplement: Supplementary file 1 [file netn-7-4-1266-s001.pdf]

Matkovič, A., Anticevic, A., Murray, J. D. & Repovš, G. (2023). Static and dynamic fMRI-derived functional connectomes represent largely similar information. *Network Neuroscience*.

Static and dynamic functional connectomes represent largely similar information

Andraž Matkovič[a,*], Alan Anticevic[b,c], John D. Murray[b,c,d], Grega Repovš[a]

[a]*Department of Psychology, Faculty of Arts, University of Ljubljana,*
[b]*Department of Psychiatry, Yale University School of Medicine, New Haven, United States,*
[c]*Interdepartmental Neuroscience Program, Yale University, New Haven, United States,*
[d]*Department of Psychiatry, Yale University, New Haven, United States,*

Supplement

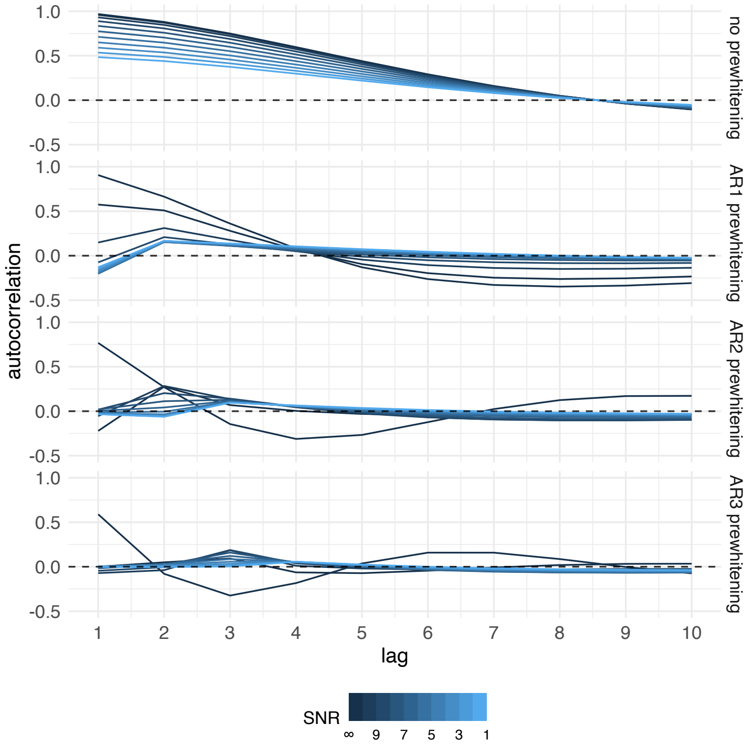

Figure S1: **The autocorrelation function of simulated data as a function of prewhitening order and noise**. The mean autocorrelation function was computed over all participants and regions. In general, noise and prewhitening reduced absolute autocorrelation. The shape of the autocorrelation function varied as a function of noise and prewhitening. In case without prewhitening, autocorrelation monotonically decreased and reached 0 at lag 8. After prewhitening, autocorrelation varied between positive and negative values, and this was most pronounced in cases without noise. The autocorrelation function was more similar to the experimental data in cases with low levels of noise.

*Corresponding author
Email address: `andraz.matkovic@ff.uni-lj.si` (Andraž Matkovič)

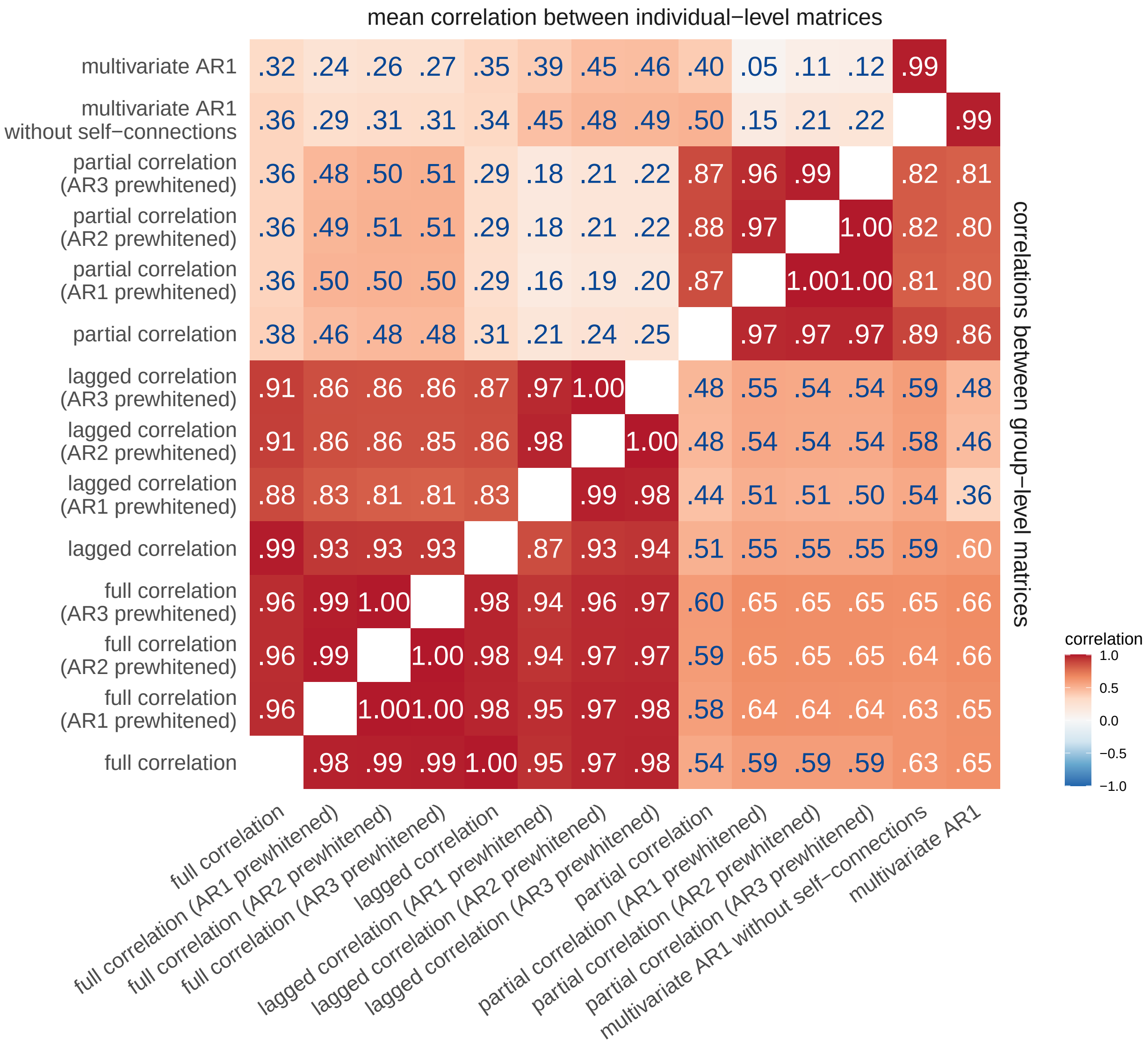

Figure S2: **Correlations between connectivity methods.** Same as in Figure 2A but includes all orders of prewhitening.

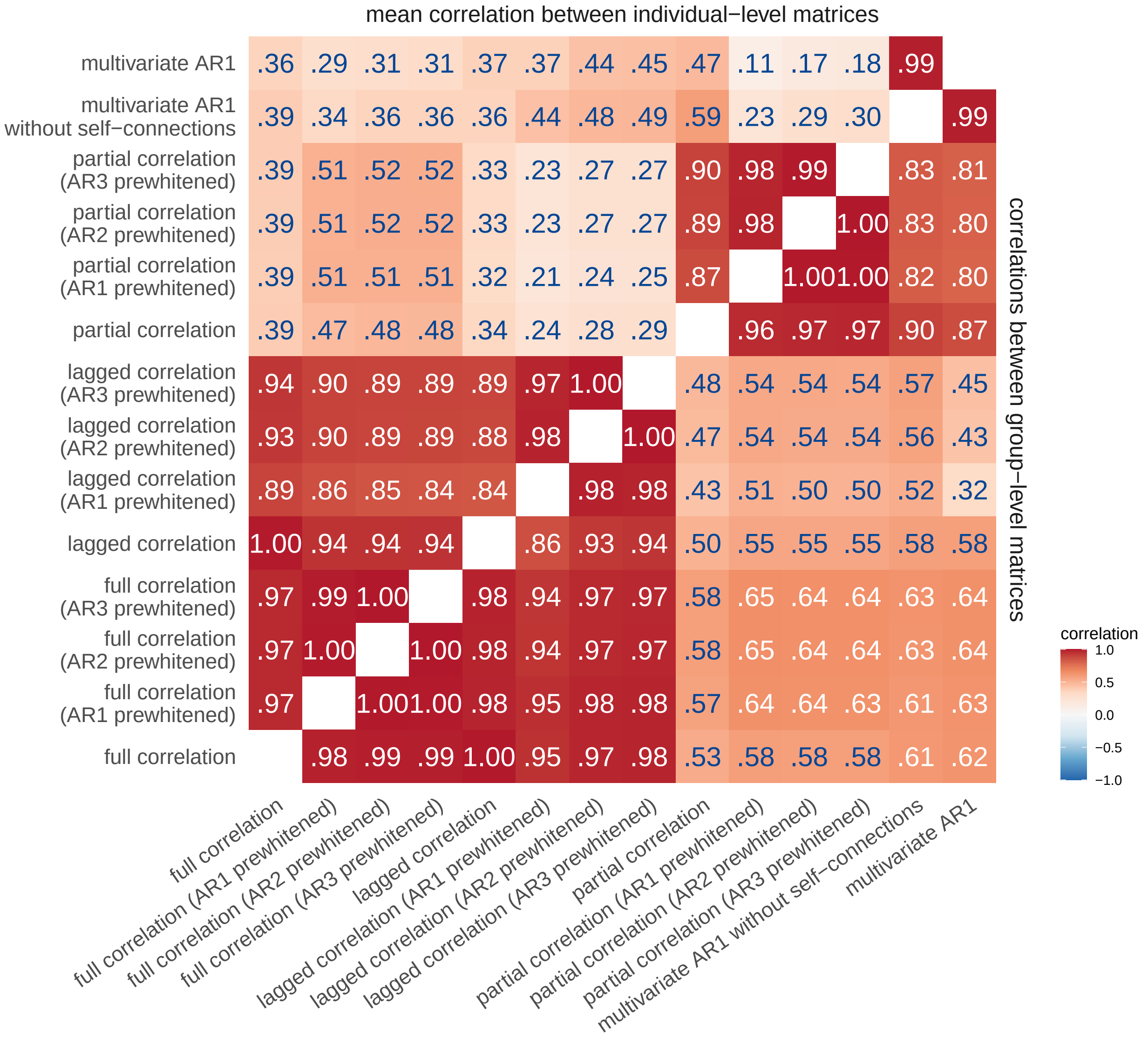

Figure S3: **Correlations between connectivity methods on 200 participants with highest quality data.**

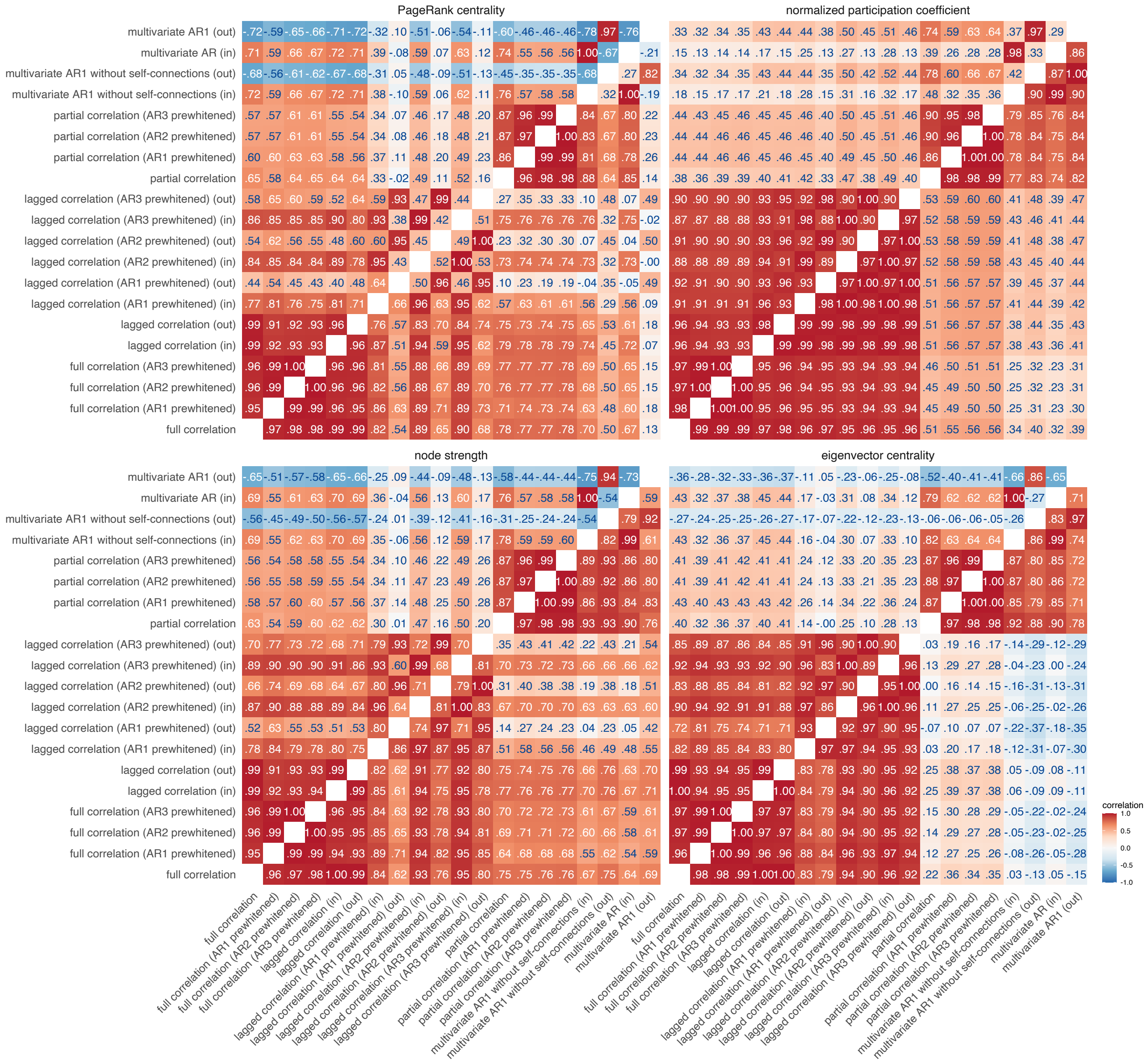

Figure S4: **Similarities between node centrality measures based on positive connections.** Similarities were estimated by (i) computing node measures on group-average connectivity matrices (group-level comparison; below diagonal), (ii) by computing node measures for each individual separately, correlating within participant and averaging these correlations across participants (individual-level comparsion; above diagonal). Same as in Figure 4, but includes prewhitened data.

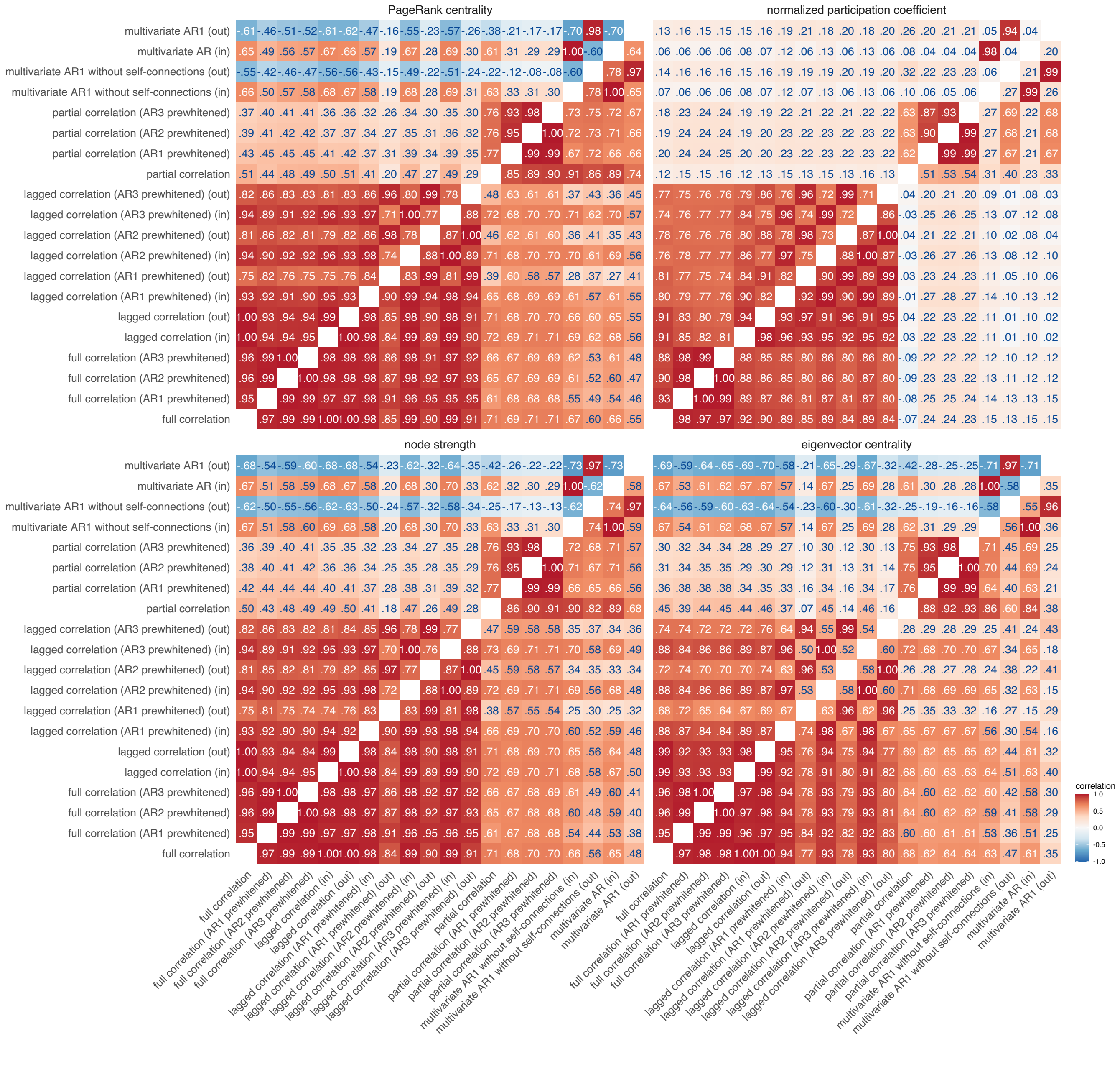

Figure S5: **Similarities between node centrality measures based on positive connections.** Similarities were estimated by (i) computing node measures on group-averaged connectivity matrices (group-level comparison; below diagonal), (ii) by computing node measures for each individual separately, correlating within participants and averaging these correlations across participants (individual-level comparison; above diagonal). Similar to Figure S4, but for negative connections.

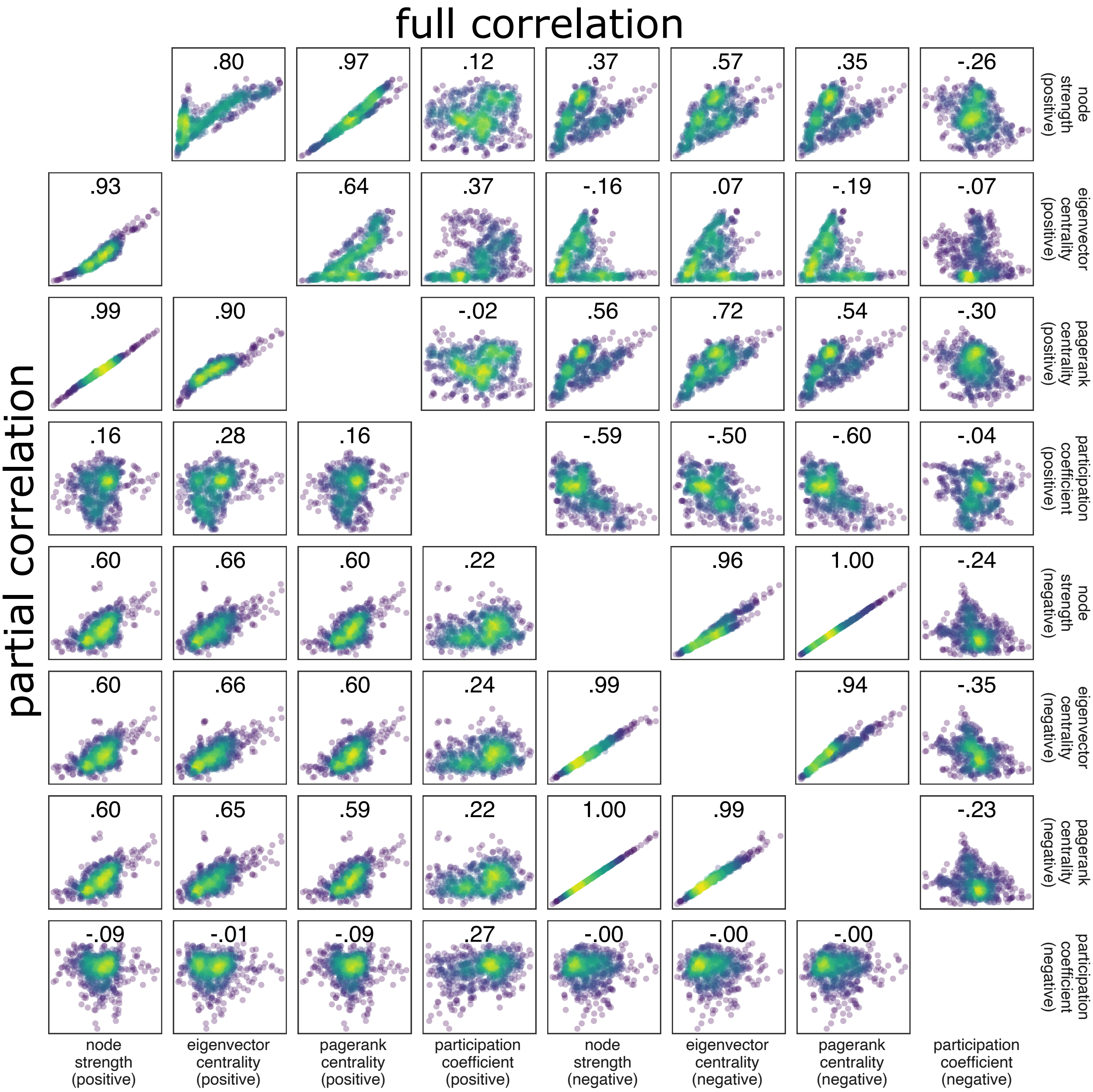

Figure S6: **Correlations between centrality measures for static FC methods at the group level.** Correlations were computed separately for positive and negative connections. We observed a positive correlation between the participation coefficient of positive connections and strength-based measures of negative connections. This suggests that nodes that participate in different modules tend to have fewer negative connections. Importantly, this finding highlights the functional importance of negative connections. However, for partial correlation networks, a positive correlation was found between strength-based measures and the participation coefficient. This suggests that indirect negative connections drive the negative relationship between participation coefficient and strength. In other words, nodes that participate in different modules tend to have more indirect negative functional connections, compared to nodes with low participation coefficient.

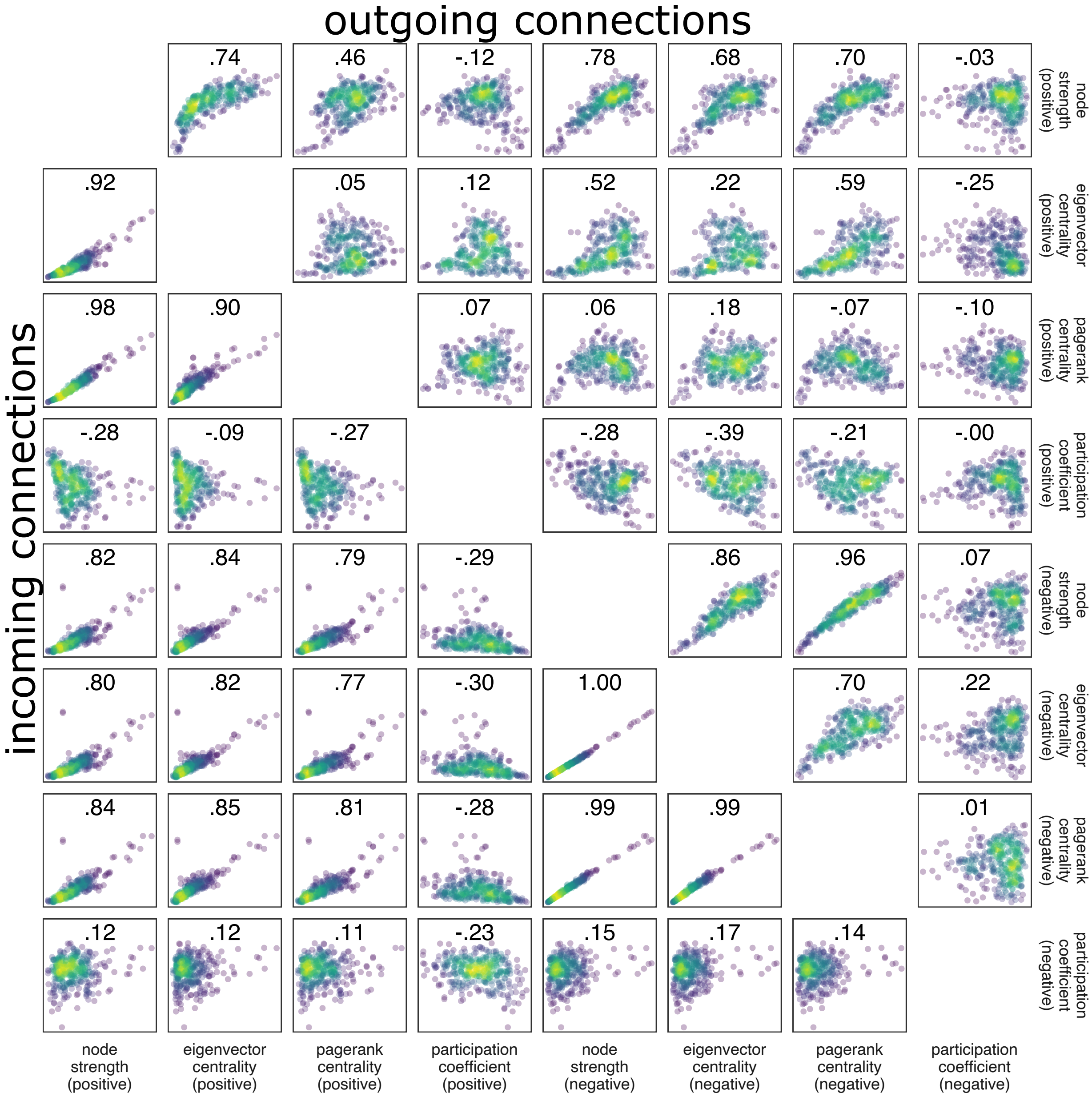

Figure S7: **Correlations between centrality measures for the multivariate autoregressive model at the group level.** Correlations were computed separately for positive and negative connections. The scatter plots above the diagonal refer to outgoing connections, while the scatter plots below the diagonal refer to incoming connections.

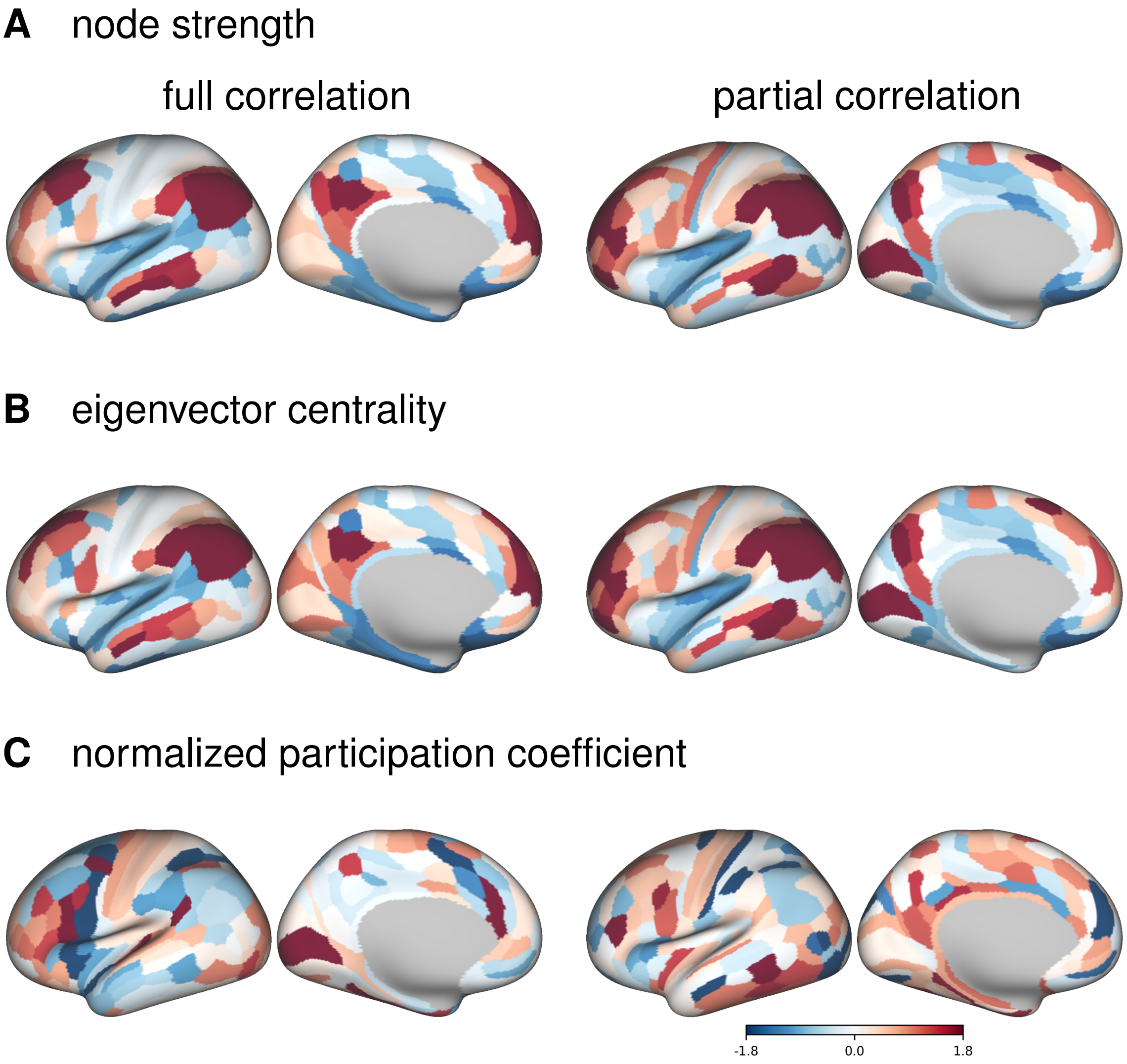

Figure S8: **Cortical distribution of centrality measures for static FC methods and for negative connections.** PageRank centrality is omitted, because its correlation with strength is equal to 1. The values have been transformed to z-values for visualization.

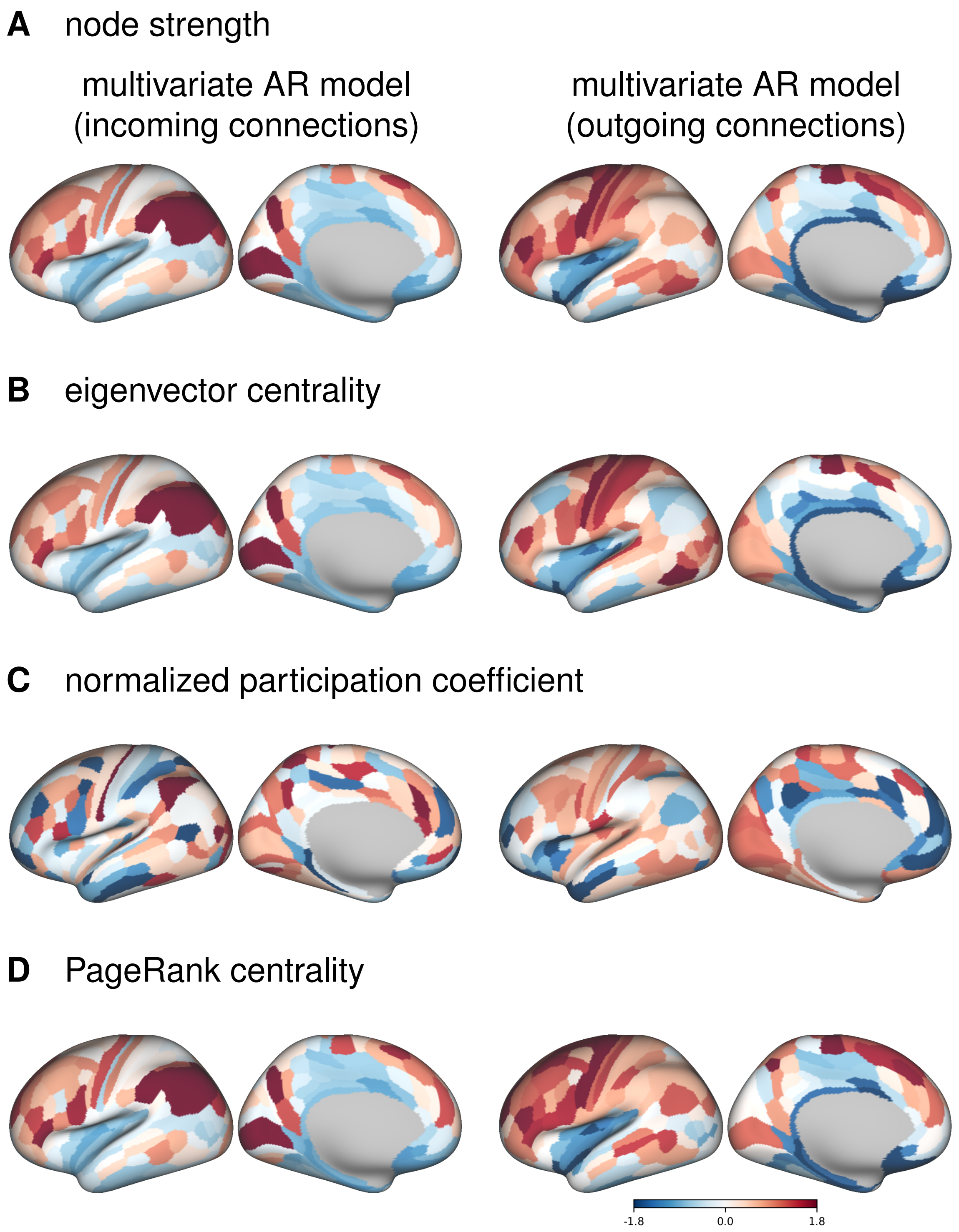

Figure S9: **Cortical distribution of centrality measures for multivariate autoregressive model and for negative connections.**

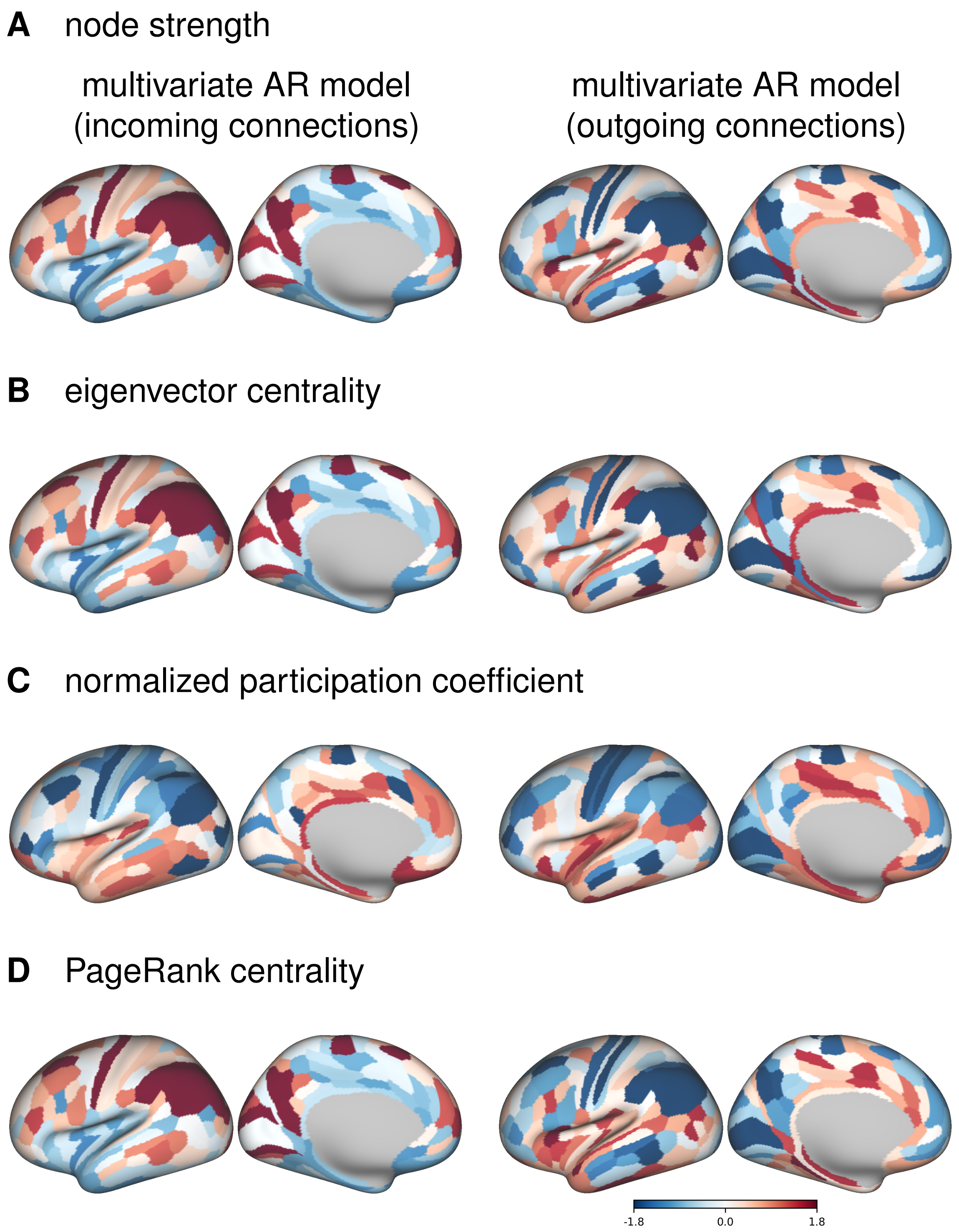

Figure S10: **Cortical distribution of centrality measures for HCP subject 100307 for multivariate autoregressive model and for negative connections.**

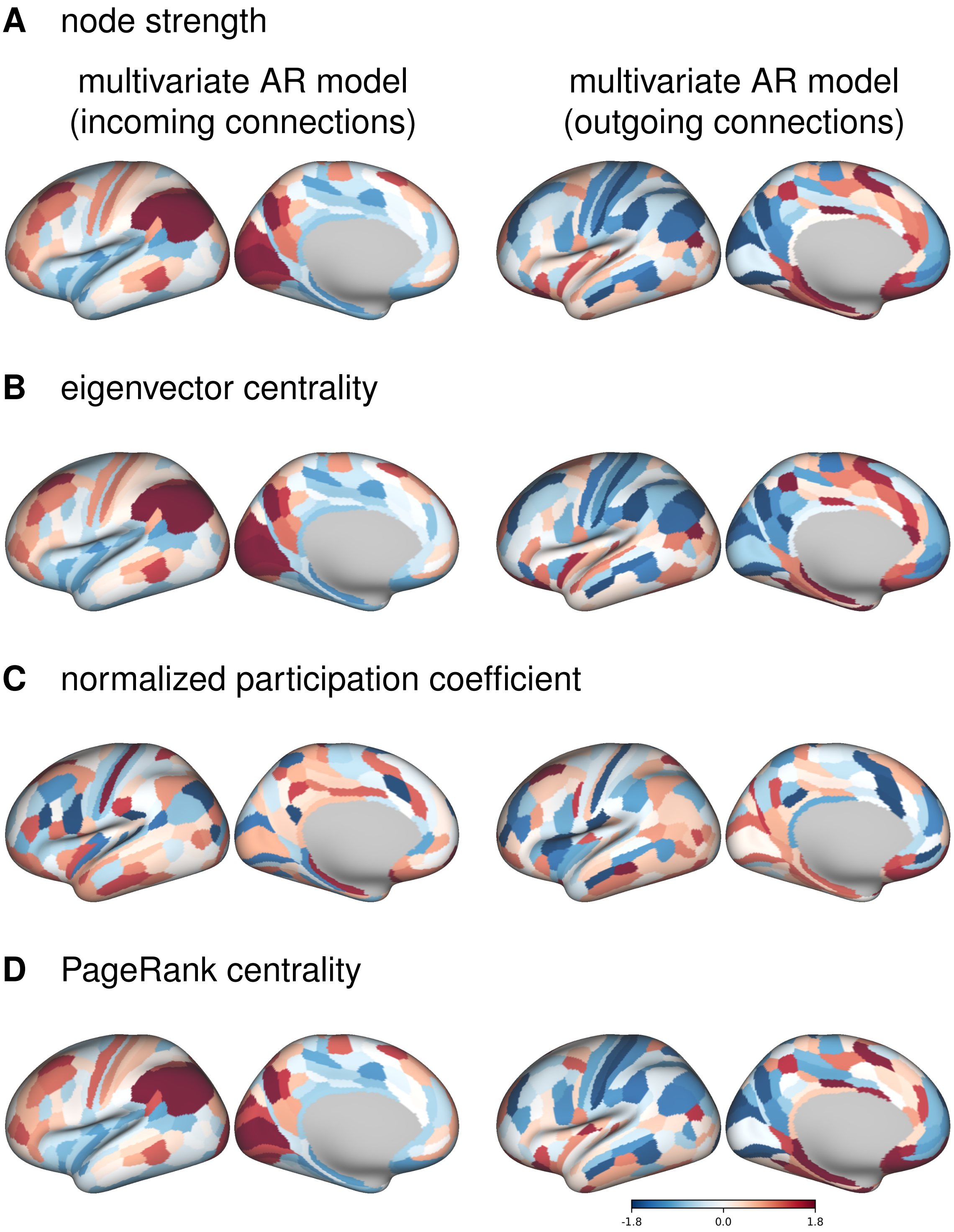

Figure S11: **Cortical distribution of centrality measures for HCP subject 100307 for multivariate autoregressive model and for negative connections.**

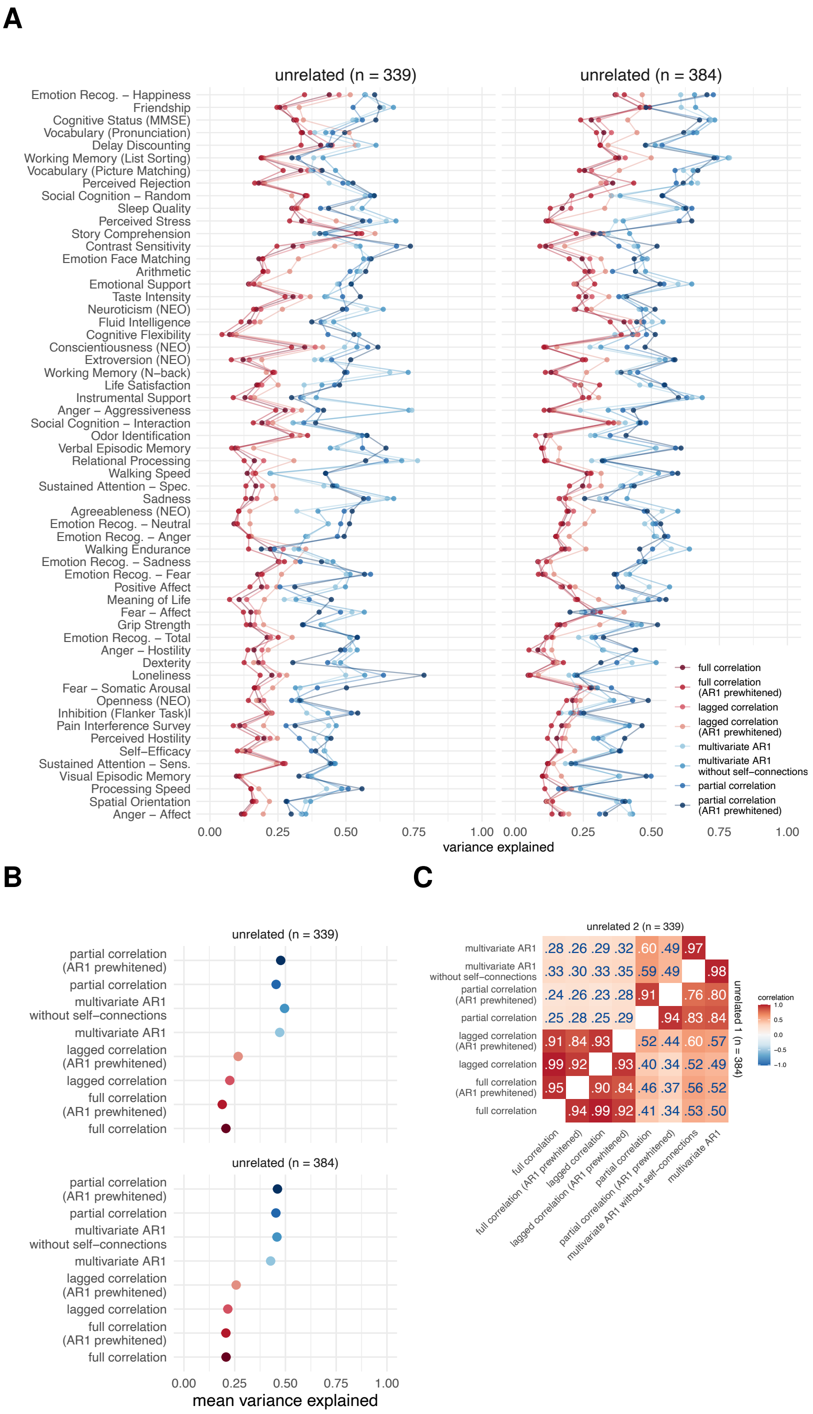

Figure S12: **Results of variance component model for brain-behavior associations on subsamples of unrelated participants.** (A) Variance explained for individual traits estimated with different connectivity methods, (B) mean variance explained, and (C) similarities of explained variance patterns between connectivity methods. The traits are ordered according to the mean variance explained across connectivity methods. The same as in Figure 7 but in subsamples of unrelated participants.

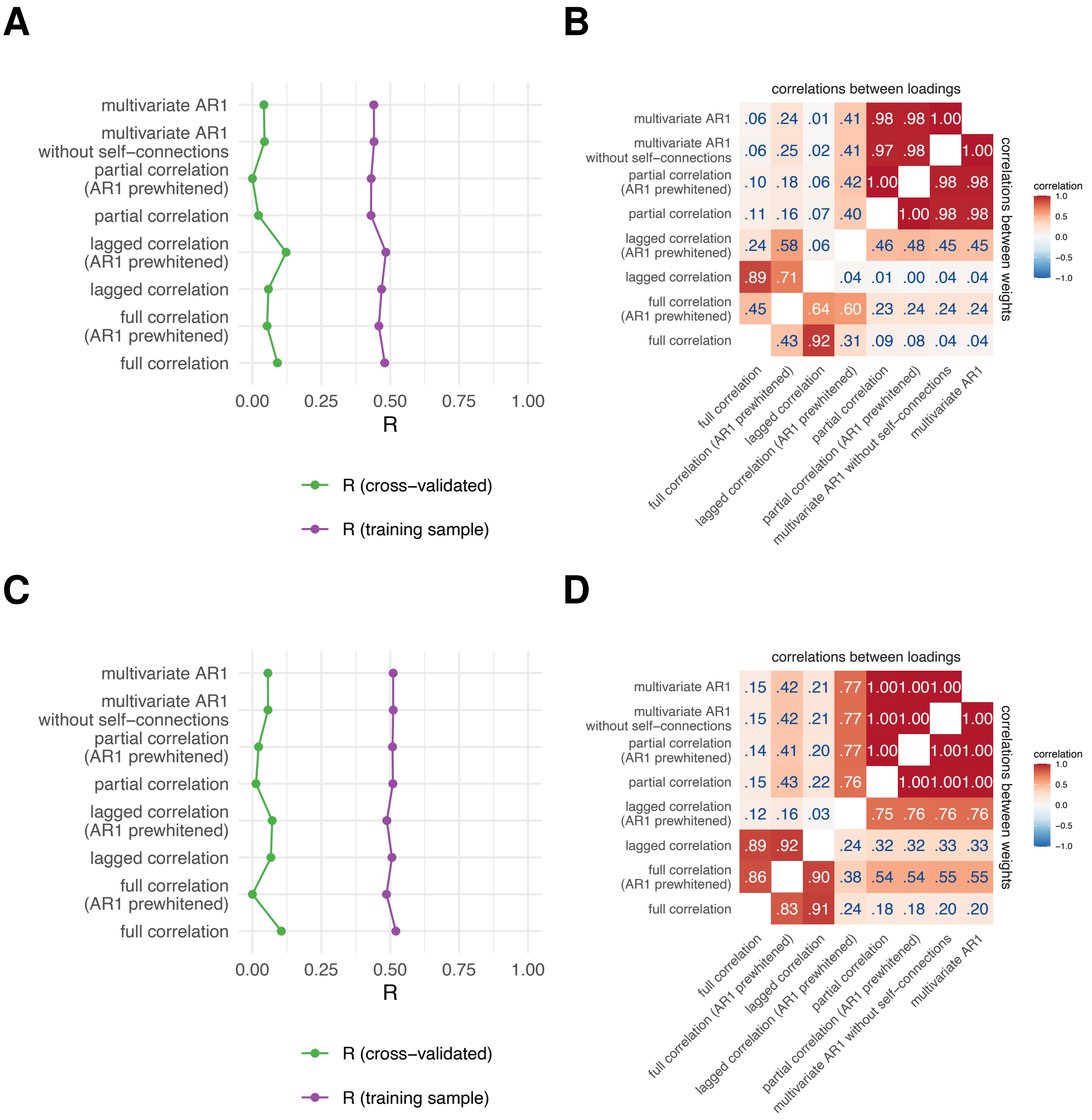

Figure S13: **Results of canonical correlation analysis for brain-behavior associations on subsamples of unrelated participants.** (A,C) First canonical correlation on test and training sets in the first (A, n = 384) and second subsample (C, n = 339). (B,D) Correlations between canonical loadings and weights across FC methods for the first canonical components on the first (B) and second (D) subsamples.

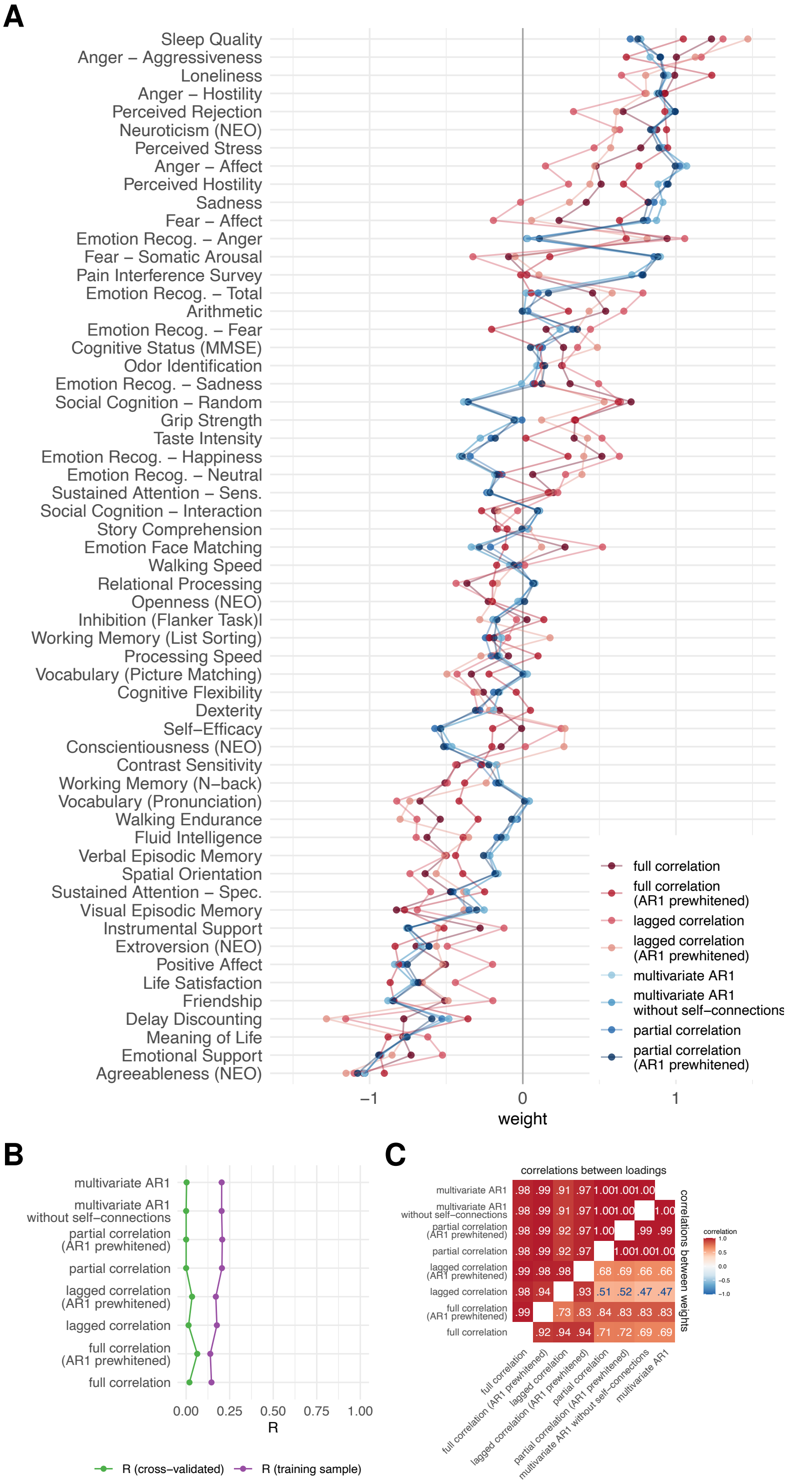

Figure S14: **Results of principal least squares analysis for brain-behavior associations. A.** PLS weights. **B.** First canonical correlation on test and training sets. **C.** Correlations between canonical loadings and weights across functional connectivity methods for first canonical components.

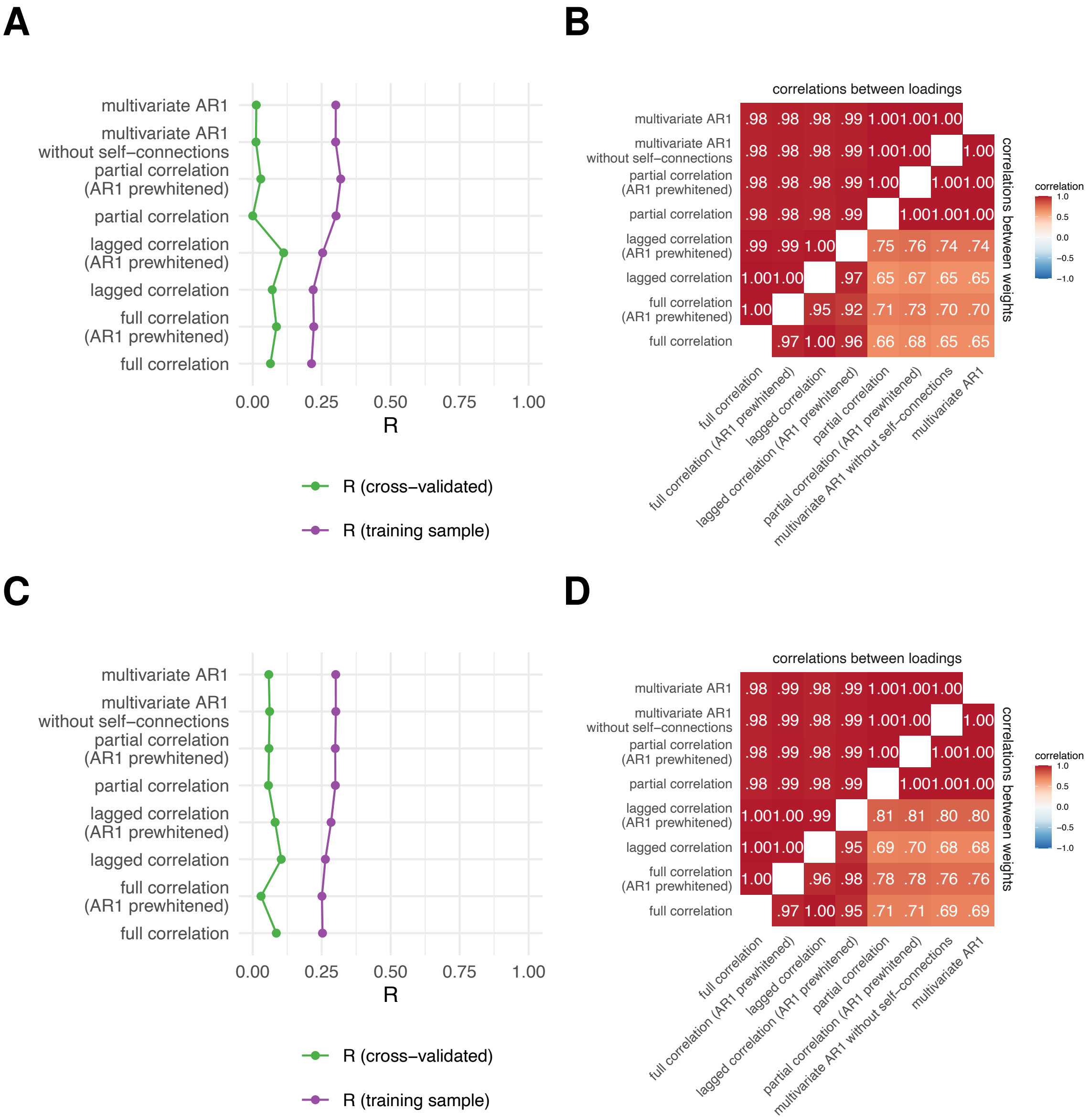

Figure S15: **Results of principal least squares analysis for brain-behavior associations on subsamples of unrelated participants.** (A,C) First canonical correlation on test and training sets in the first (A, n = 384) and second subsample (C, n = 339). (B,D) Correlations between canonical loadings and weights across FC methods for the first canonical components on the first (B) and second (D) subsamples.

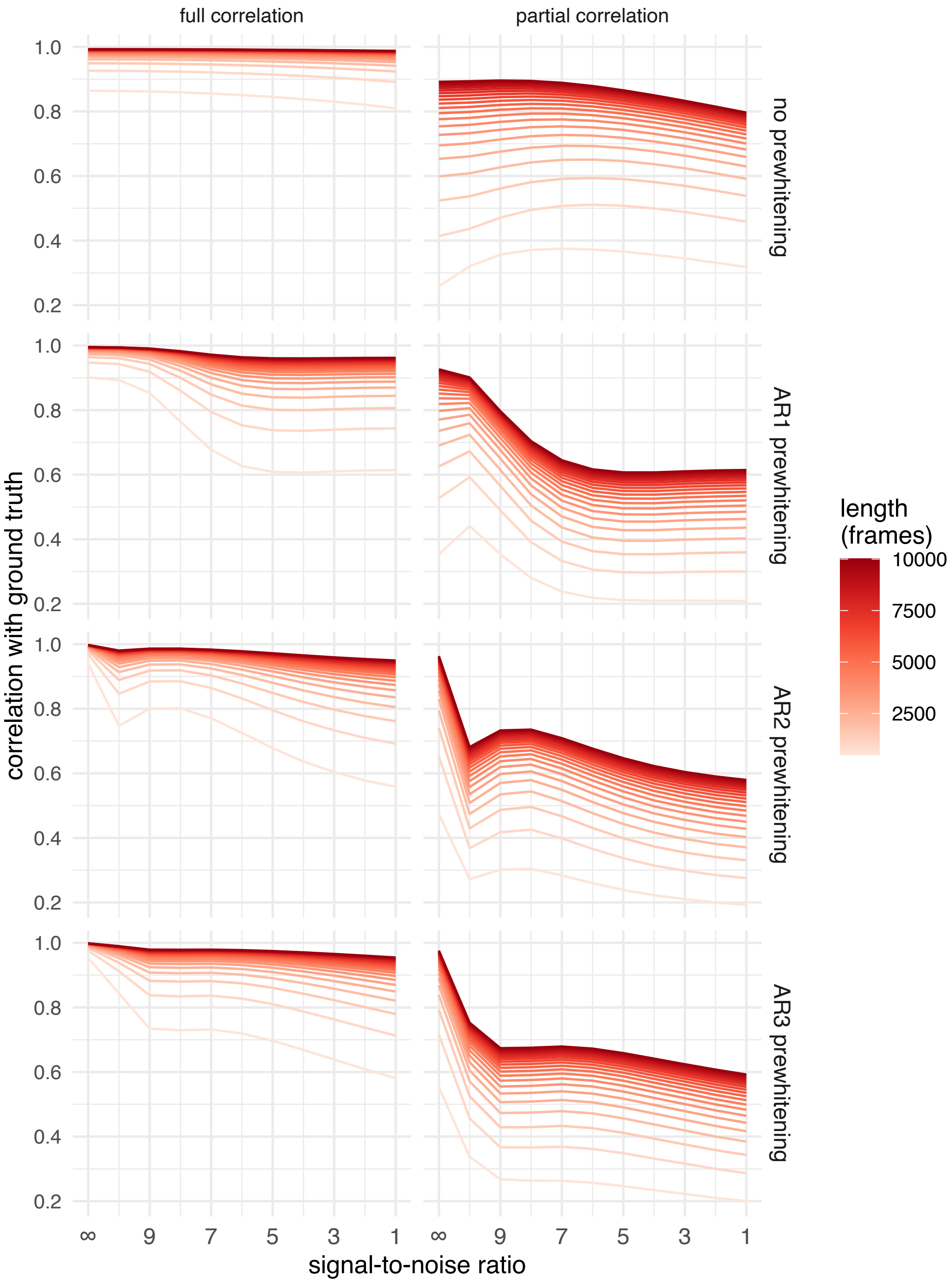

Figure S16: **Correlation between ground truth and simulated data for all FC methods in association ith noise and signal length.** Same as in Figure 11B but includes all orders of prewhitening.

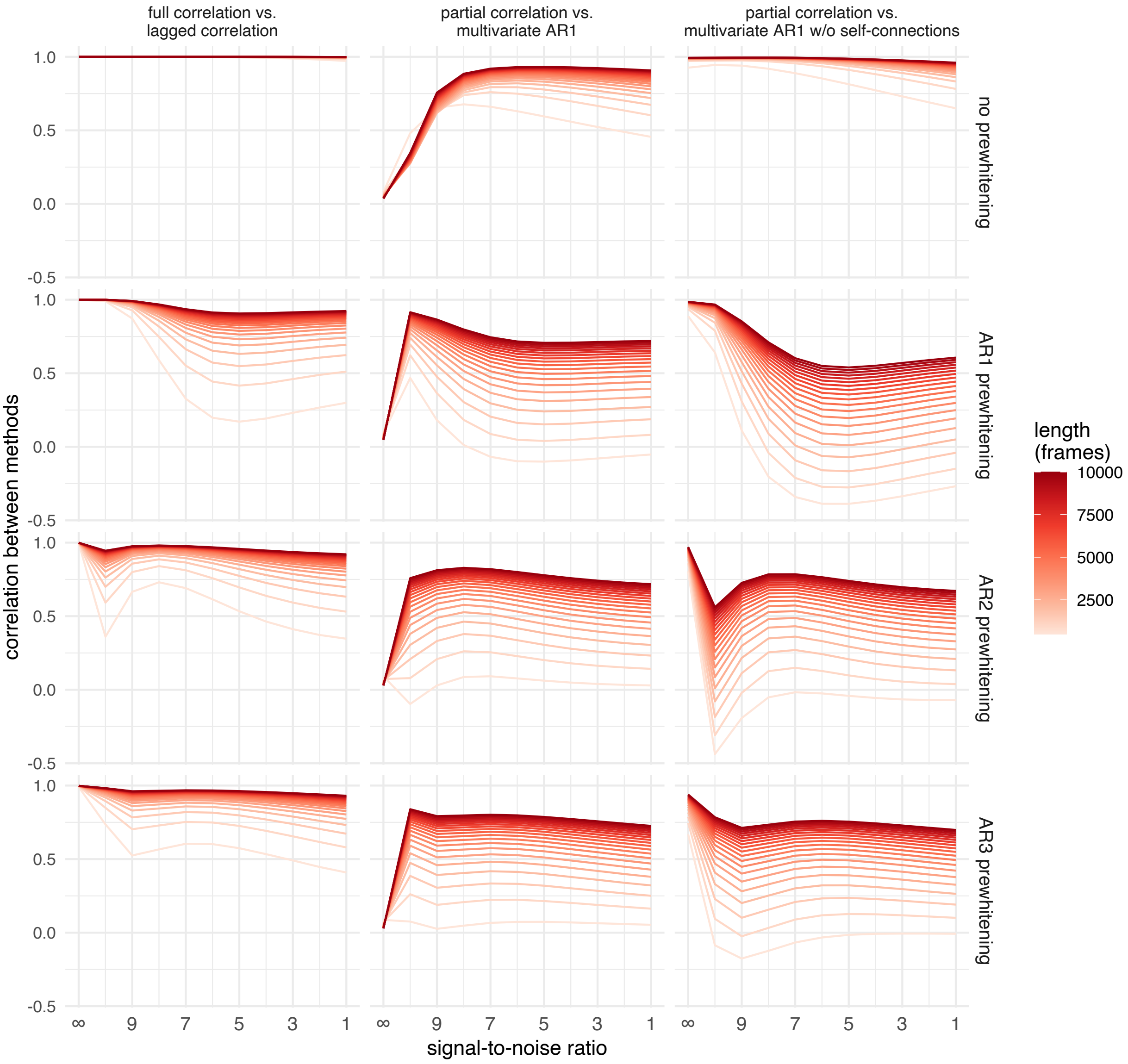

Figure S17: **Correlation between selected pairs of FC methods as a function of noise and signal length on simulated data.** Same as in Figure 11C but includes all prewhitening orders.

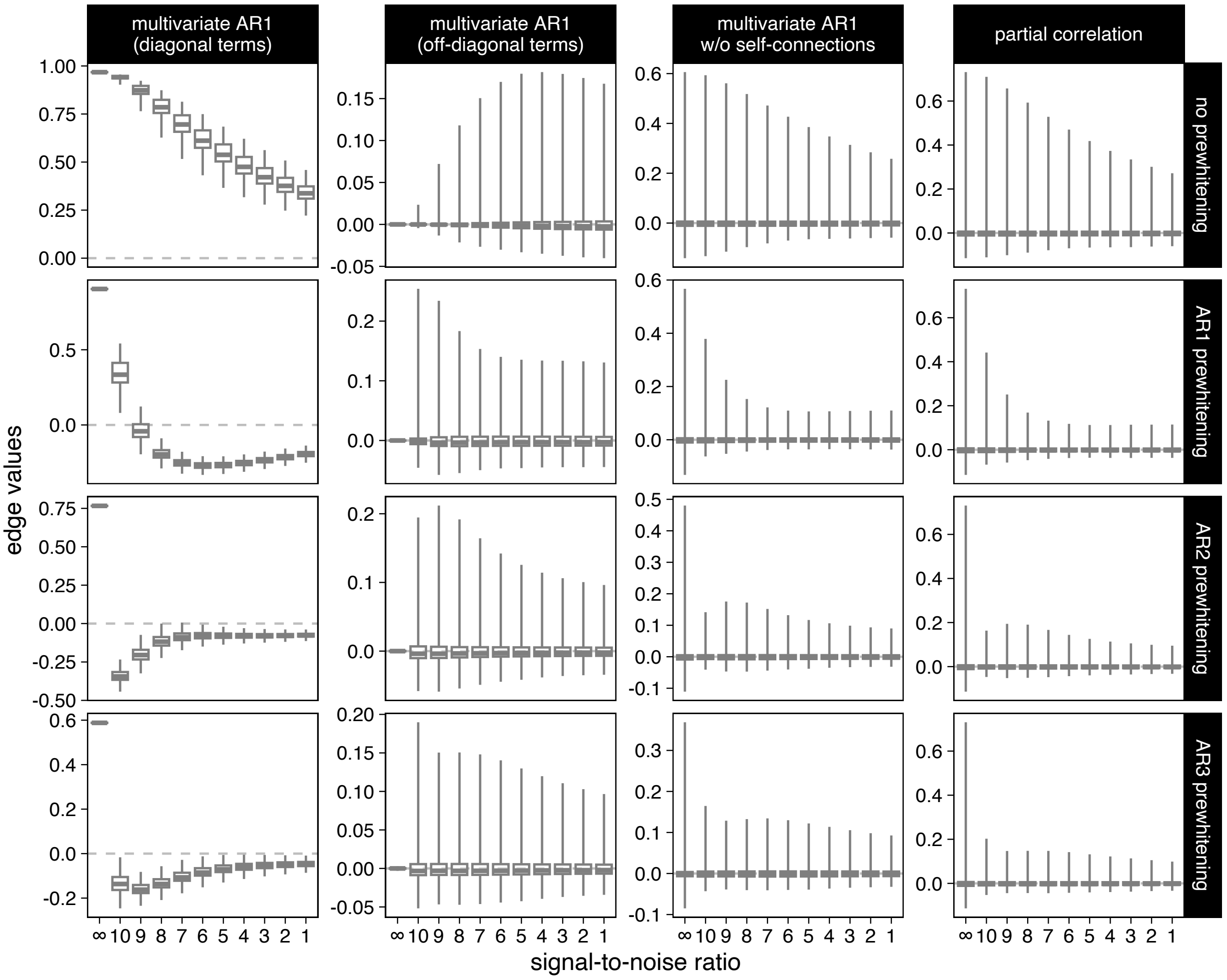

Figure S18: **Distributions of edge values on simulated data for selected FC methods as a function of noise for the signals with the longest length (10000 frames).** The distributions are based on the average FC matrix across simulated participants. The boxplot whiskers represent the minimum and maximum values.

HCP Field	Friendly Name	HCP Field	Friendly Name
PicSeq_Unadj	Visual Episodic Memory	WM_Task_Acc	Working Memory (N-back)
CardSort_Unadj	Cognitive Flexibility	NEOFAC_A	Agreeableness (NEO)
Flanker_Unadj	Inhibition (Flanker Task)	NEOFAC_O	Openness (NEO)
PMAT24_A_CR	Fluid Intelligence	NEOFAC_C	Conscientiousness (NEO)
ReadEng_Unadj	Vocabulary (Pronunciation)	NEOFAC_N	Neuroticism (NEO)
PicVocab_Unadj	Vocabulary (Picture Matching)	NEOFAC_E	Extroversion (NEO)
ProcSpeed_Unadj	Processing Speed	ER40_CR	Emotion Recog. - Total
DDisc_AUC_40K	Delay Discounting	ER40ANG	Emotion Recog. - Anger
VSPLOT_TC	Spatial Orientation	ER40FEAR	Emotion Recog. - Fear
SCPT_SEN	Sustained Attention - Sens.	ER40HAP	Emotion Recog. - Happiness
SCPT_SPEC	Sustained Attention - Spec.	ER40NOE	Emotion Recog. - Neutral
IWRD_TOT	Verbal Episodic Memory	ER40SAD	Emotion Recog. - Sadness
ListSort_Unadj	Working Memory (List Sorting)	AngAffect_Unadj	Anger - Affect
MMSE_Score	Cognitive Status (MMSE)	AngHostil_Unadj	Anger - Hostility
PSQI_Score	Sleep Quality	AngAggr_Unadj	Anger - Aggressiveness
Endurance_Unadj	Walking Endurance	FearAffect_Unadj	Fear - Affect
GaitSpeed_Comp	Walking Speed	FearSomat_Unadj	Fear - Somatic Arousal
Dexterity_Unadj	Dexterity	Sadness_Unadj	Sadness
Strength_Unadj	Grip Strength	LifeSatisf_Unadj	Life Satisfaction
Odor_Unadj	Odor Identification	MeanPurp_Unadj	Meaning of Life
PainInterf_Tscore	Pain Interference Survey	PosAffect_Unadj	Positive Affect
Taste_Unadj	Taste Intensity	Friendship_Unadj	Friendship
Mars_Final	Contrast Sensitivity	Loneliness_Unadj	Loneliness
Emotion_Task_Face_Acc	Emotion Face Matching	PercHostil_Unadj	Perceived Hostility
Language_Task_Math_Avg_Difficulty_Level	Arithmetic	PercReject_Unadj	Perceived Rejection
Language_Task_Story_Avg_Difficulty_Level	Story Comprehension	EmotSupp_Unadj	Emotional Support
Relational_Task_Acc	Relational Processing	InstruSupp_Unadj	Instrumental Support
Social_Task_Perc_Random	Social Cognition - Random	PercStress_Unadj	Perceived Stress
Social_Task_Perc_TOM	Social Cognition - Interaction	SelfEff_Unadj	Self-Efficacy

Table S1: Behavioral measures.
